# Discovery of Gastric Metastases From Primary Renal Cell Carcinoma Through MRI: A Case Report

**DOI:** 10.7759/cureus.43637

**Published:** 2023-08-17

**Authors:** Adrien Saifi, Christiane Jungels, Ana Veron Sanchez

**Affiliations:** 1 Radiology, Institut Jules Bordet, Brussels, BEL; 2 Oncology, Institut Jules Bordet, Brussels, BEL

**Keywords:** clear cell renal cell carcinoma, stomach, renal cell carcinoma, gastric metastases, gastroenterology, oncology, magnetic resonance imaging, nuclear magnetic resonance, radiology

## Abstract

Gastric metastases from primary renal cell carcinoma (RCC) are rare and poorly documented in the existing literature. This case report presents the clinical course of a 65-year-old male with multi-metastatic clear cell RCC (ccRCC) who was incidentally found to have stomach metastases during follow-up magnetic resonance imaging (MRI). Gastric metastases from ccRCC are typically associated with other metastatic sites. They often emerge at an advanced stage of the disease, indicating a poor prognosis. It is therefore important to consider gastric metastases as a potential site of involvement in RCC patients. MRI revealed three gastric mucosal lesions exhibiting hypervascularity, a characteristic feature of ccRCC. Histological analysis confirmed the presence of malignant cells compatible with RCC.

## Introduction

Renal cell carcinoma (RCC) represents over 3% of all cancers, with a male predominance (male: female 2:1) [1]. The most common types of renal carcinoma are clear cell, papillary, and chromophobe, together accounting for 85% of cases [2]. During the time of detection, more than a quarter of cases display an advanced condition, either in a localized or metastatic stage [3]. The most frequent metastatic sites are the lungs (45.2%), bone (29.5%), lymph nodes (21.8%), liver (20.3%), and adrenal glands (8.9%) [4]. However, metastases may involve any organ in the body.

Gastric metastases from RCC are exceedingly rare and have limited documentation in existing literature [5]. The prognosis for patients with gastric metastases is generally unfavorable [6]. These cases are nearly always found in conjunction with other metastases during diagnosis. Additionally, they tend to manifest in the advanced stages of the disease, typically occurring around 6.9 years (with a range of 1.7 to 13.1 years) after the initial RCC diagnosis [6]. In this report, we present the case of a patient presenting with multi-metastatic clear cell RCC (ccRCC), who was incidentally discovered to have gastric metastasis during follow-up magnetic resonance imaging (MRI).

## Case presentation

The patient was a 65-year-old Italian male who lived in Belgium (Europe). He was a non-smoker and had ischemic cardiomyopathy and hypothyroidism. He was diagnosed with right ccRCC in February 2014 and underwent a total right nephrectomy. In April 2014, he experienced a non-operable relapse characterized by right pulmonary hilar adenopathy and left upper lobe lung metastasis. Over the course of 2014 to 2022, he underwent several targeted chemotherapy treatments and immunotherapy, leading to the achievement of stable disease status. In 2022, the disease demonstrated progression marked by the presence of multiple nodules of peritoneal carcinomatosis and abdominal lymphadenopathies. The patient presented no symptoms related to this progression. The physical examination did not reveal any noteworthy findings. The last blood test indicated the presence of anemia with a hemoglobin level of 9.7 g/dl and hypoalbuminemia at 27 g/l, persisting for the past three months.

**Figure 1:**
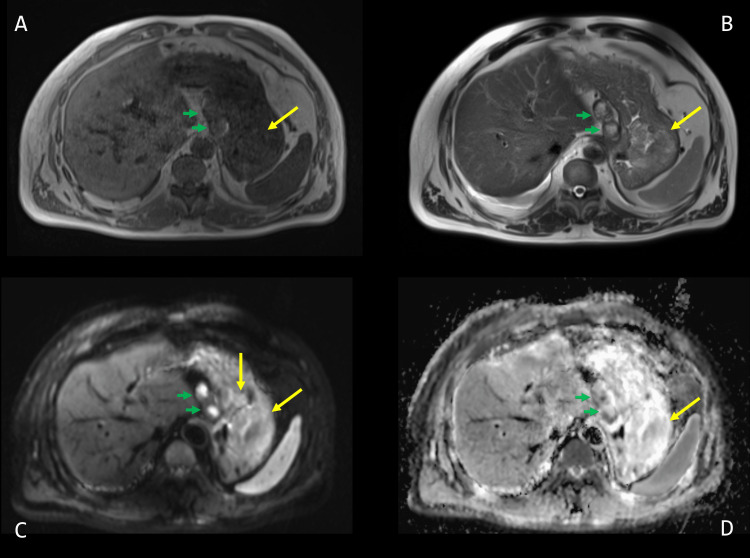
Axial T1-weighted (A), T2-weighted (B), and diffusion-weighted (C) magnetic resonance imaging of the abdomen with apparent diffusion coefficient (D) Two gastric mucosal lesions (yellow arrows) appear as polypoid masses protruding into the lumen of the gastric fundus and body. They appear hypointense on the T1-weighted image (A) and slightly hyperintense on the T2-weighted image (B). They show no significant diffusion restriction on diffusion-weighted imaging (C) and apparent diffusion coefficient (D). In addition, two necrotic adenopathies (green arrows) are visible against the lesser curvature of the stomach.

**Figure 2:**
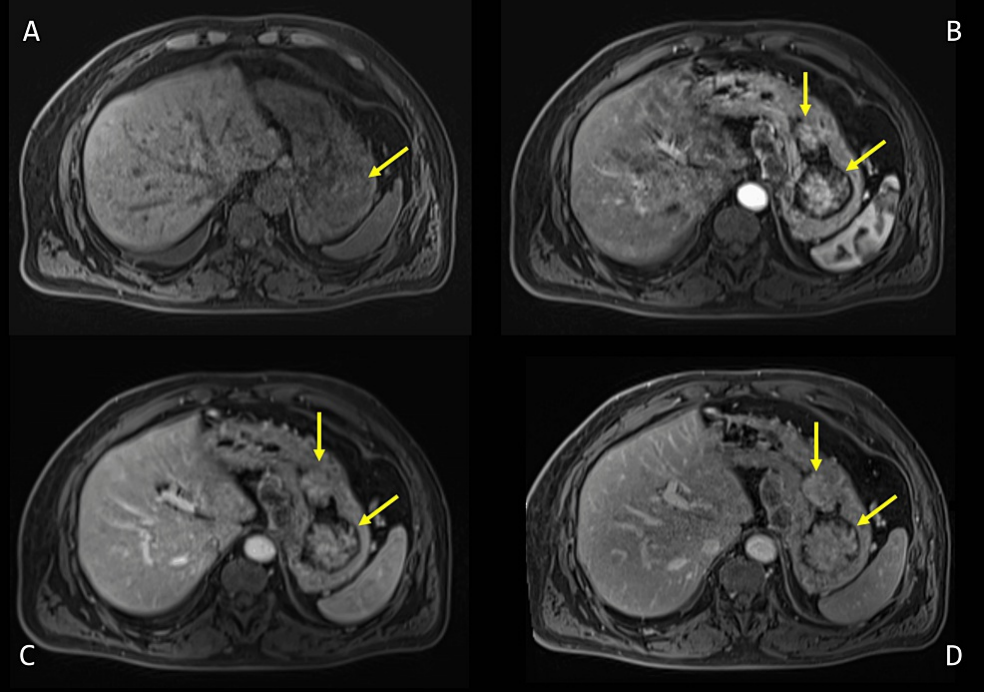
Axial fat-saturated unenhanced T1-weighted (A) and axial fat-saturated contrast-enhanced T1-weighted magnetic resonance imaging of the abdomen on arterial phase (B), portal phase (C) and three-minute late venous phase (D) The gastric lesions (yellow arrows) appear hypointense on the fat-saturated T1-weighted image (A). They exhibit the characteristic hypervascular behavior of clear cell renal cell carcinoma, demonstrating intense early heterogeneous enhancement during the arterial phase (B), progressing during the portal venous phase (C) and the three-minute late venous phase (D).

During his follow-up, only computed tomography (CT) scans had been performed, and the patient had a gadolinium-enhanced abdominal MRI four months after the last abdominal CT scan to characterize the tumor progression (Figures 1-2). This MRI clearly showed the presence of three gastric mucosal lesions, which appeared as polypoid masses protruding into the lumen of the fundus and the body of the stomach. They were hypointense on the T1-weighted image and slightly hyperintense on the T2-weighted image (Figure 1). On diffusion-weighted imaging, the lesions showed no significant diffusion restriction (Figure 1). They exhibited the characteristic hypervascular behavior of ccRCC, demonstrating intense early heterogeneous enhancement during the arterial phase, progressing during the portal venous phase and three-minute late venous phase (Figure 2).

**Figure 3:**
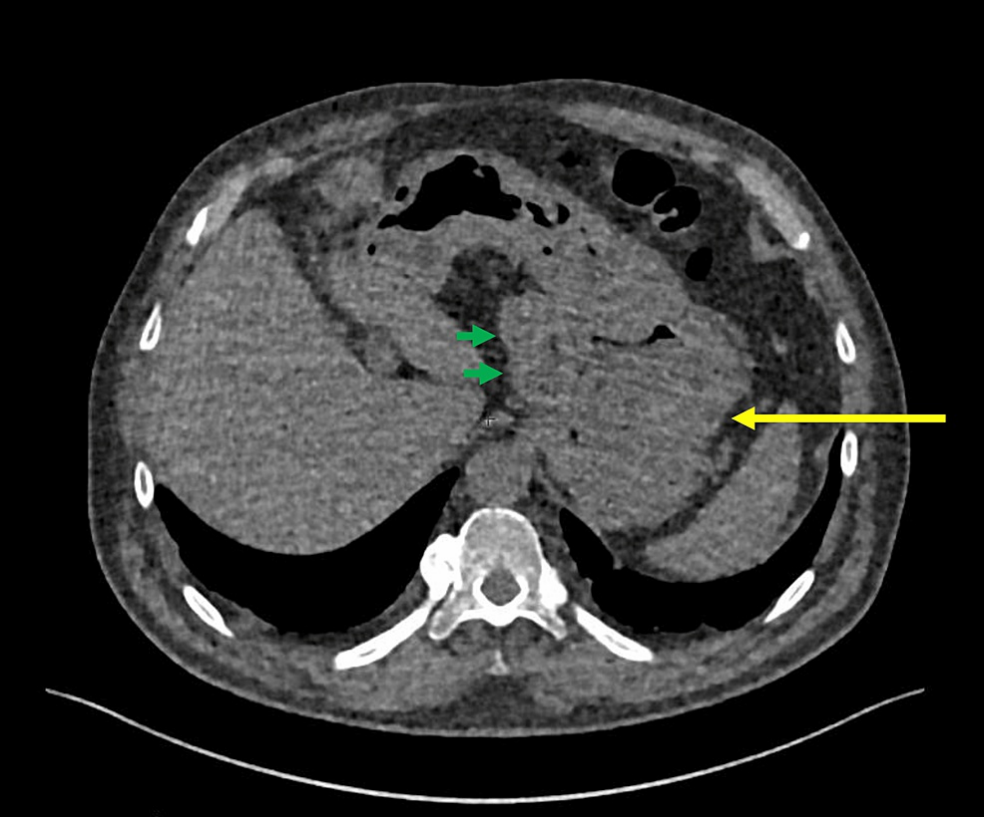
Lower section of unenhanced chest CT Retrospectively, the gastric metastases (yellow arrows) could have been only suspected in the lower sections of the last follow-up unenhanced chest CT, which was performed one month before the MRI. However, the CT was incapable of definitively diagnosing the condition. Moreover, the two necrotic adenopathies (green arrows) were clearly visible against the lesser curvature of the stomach.

**Figure 4:**
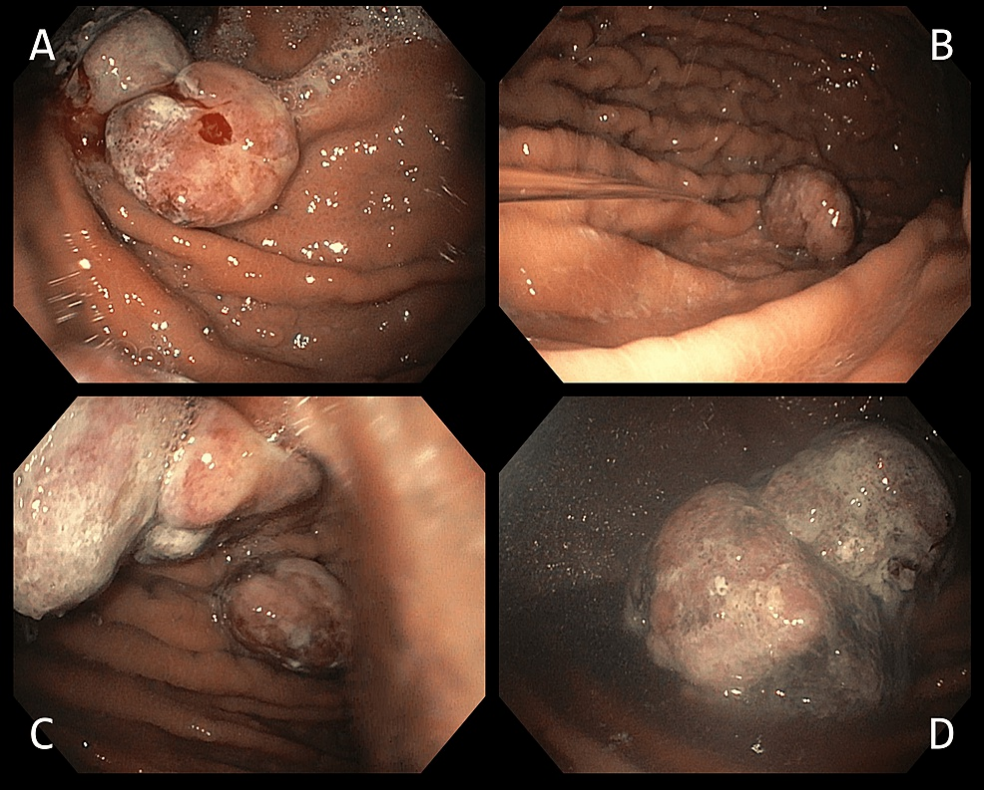
Gastroscopy demonstrating two lesions in the gastric fundus and one in the greater curvature of the body Even though the lesions had a rich blood supply, there was no evidence of any bleeding.

Retrospectively, the potential existence of gastric metastases could have been only suspected in the lower sections of the last follow-up unenhanced chest CT, which was performed one month before the MRI (Figure 3). However, it was the MRI that provided the evidence, rendering the suspicion of metastases only possible after its results. The CT was incapable of definitively diagnosing the condition. A supplementary biopsy by gastroscopy and histological exam were performed following the MRI. During the gastroscopy, two lesions were found in the fundus and one in the greater curvature of the body, confirming the MRI scan results (Figure 4). None of the lesions was bleeding at the time of examination despite their highly vascular appearance. Several samples were taken for histological analysis.

The histological examination revealed the presence of ulceration, characterized by a fibrin-leukocyte exudate containing cells with round discreetly irregular nuclei. Supplementary histochemical examinations revealed a strongly positive result for vimentin, AE1 and AE3 (pancytokeratins), as well as CD10 (proximal tubular epithelial marker) immunostaining. These results confirmed the presence of malignant cells compatible with renal cell carcinoma.

## Discussion

RCC stands as the prevailing solid lesion found in the kidney [1]. This type of cancer encompasses various subtypes, each presenting distinct histopathological characteristics. Among these subtypes, ccRCC emerges as the most frequently encountered [1]. This subtype also carries a less favorable prognosis when compared to other histopathological entities [1].

Gastric metastases are rare, mostly associated with malignant melanoma, lung and breast carcinomas [5,7]. Stomach involvement in metastatic RCC is extremely rare, except when invaded by metastases in neighboring organs [8,9]. It is for this reason that there is very little scientific literature dealing with this kind of metastasis; only about 50 cases are reported [10]. The purpose of this case report is to remind readers to always consider this potential metastatic site, given the lack of scientific literature dealing with its subject. Besides, gastric metastases are usually asymptomatic and discovered as incidental findings on cross-sectional imaging. If symptomatic, acute or chronic gastrointestinal bleeding and anemia may occur due to the lesion's hypervascularity [5,8]. Symptoms associated with tumor bleeding are estimated to occur in 81.8% of metastases from RCC as the first symptom [11].

Malignant spread of RCC to the stomach is not common at the time of diagnosis of RCC but appears much later in the history of the disease [6,11]. Namikawa et al. observed that the median time interval between complete resection of the primary tumor and diagnosis of gastric metastasis was 6.3 years (ranging from 1 to 23 years) [11]. In addition, the prognosis of patients who develop gastric metastasis during these first 6.3 years is significantly worse than patients who develop it later [11]. Indeed, the median survival of these patients is five months before 6.3 years and 24 months after this time [11]. In general, its presence is indicative of a poor prognosis with a median survival of 19 months [11].

In the event of a solitary gastric metastasis with feasible surgical treatment, resection is the best option to achieve significant survival prolongation [8]. Although the risk of recurrence is significant, this approach enables a delay in the start of systemic treatment, regardless of whether the resection is done endoscopically or surgically [8]. Moreover, it is an efficient and rapid solution if the metastasis causes acute bleeding [8]. If multiple, complete resection of metastases also increases patient survival [12,13].

Moreover, gastric metastases from RCC have multiple macroscopic appearances [11,14]. There is no typical appearance allowing them to be identified directly by eye during gastroscopy [11]. According to Kim et al., polypoid morphology is the most frequent (50.6%) but this type of stomach metastases can also take the form of an ulcer (17.7%), relatively large mass (10.1%), elevated lesion (10.1%), or minor erosion (3.8%) [14]. On imaging, these metastases usually appear as a well-circumscribed, intragastric polypoid mass [14]. They are mostly heterogeneous on unenhanced T1-weighted images and hyperintense on T2-weighted images [15]. They have a behavior equivalent to ccRCC after intravenous injection of gadolinium, namely, a strong heterogeneous enhancement on the arterial phase and a late wash-out [14].

RCC metastatic sites are very numerous due to the multitude of routes that the metastases can take: hematogenous, lymphogenous, renal capsule, renal pelvis, and ureteric routes [8,9]. The hematogenous route is the most frequent due to the richly vascularized character of RCC [16]. The systematic review of Prudhomme et al. indicates that the most frequent locations of this type of metastasis are as follows: the body of the stomach (45%), fundus (36%), and pylorus (18%) [17]. Moreover, they are more likely to be unique than multiple [5].

## Conclusions

In conclusion, gastric metastases from primary renal cell carcinoma (RCC) are rare and often found in conjunction with other metastases during diagnosis. They usually manifest in the advanced stages of the disease, several years after the initial RCC diagnosis, indicating a poor prognosis. Considering that gastric metastases are often discovered incidentally, it is essential to search for them in cross-sectional imaging examinations during RCC follow-up to ensure early detection and adapted patient management.
